# Strategies for Generating Footsteps of Biped Robots in Narrow Sight

**DOI:** 10.3390/s22103817

**Published:** 2022-05-18

**Authors:** Sung-Joon Yoon, Baek-Kyu Cho

**Affiliations:** 1 Department of Mechanical Systems Engineering, Kookmin University, Jeongneung-ro 77, Seoul 02707, Korea; densee250@gmail.com; 2 School of Mechanical Engineering, Kookmin University, Jeongneung-ro 77, Seoul 02707, Korea

**Keywords:** path planning, point cloud, humanoid, legged robot

## Abstract

In this paper, we present a strategy for a legged robot to stably cross cinder blocks with a limited area acquired from a camera. First, we used the point cloud acquired from the camera to detect the planes and calculate their centroids and directions. This information was used to determine the position and direction of the foot to which the robot should go. Existing A*-based footstep planners require a global map to reach the goal from the start and do not generate a path if there is no solution to the goal due to completeness of A*. In addition, if the map is not updated while moving the path, it is vulnerable to changes in the object position. Our strategy calculates the footsteps that the robot can walk in a limited camera area without securing a global map. In addition, it updates the local map information every walking step so that it quickly recognizes nearby objects and finds a path that can move. While the robot is walking, objects may not be detected due to the narrow camera field of view. In addition, even if an area for the robot to land is found, a situation in which the robot’s legs collide may occur. We present a strategy to solve this problem using previous landing data. In the experimental environment composed of several patterns, the performance was verified by stably walking on the blocks without collision between the robot’s legs.

## 1. Introduction

With the advancement of robot technology, many robots have been developed to perform tasks that are dangerous for humans to work. For example, when Japan’s Fukushima nuclear power plant exploded, wheeled robots were used as robots to withstand radiation and perform exploration missions [1]. These wheeled robots move quickly but have limited movement in uneven terrain and obstacle environments. This movement problem can be solved with a legged robot. A legged robot can cross obstacles by walking or jumping, and has the advantage of walking on uneven terrain. Recent studies suggest a slip detection approach to move on unstructured terrain [2] or a reconfiguration method of a small quadruped robot into multi-legged robot swarms to move on rough terrain [3]. In order for such a legged robot to reach a goal, it is necessary not only to use the walking control technique but also to recognize the terrain and find footsteps that can move to the destination. There are various strategies for footstep selection. For example, there are methods of generating footstep reflecting joystick input commands [4], placing footstep controlling system energy for stable walking [5], and generating autonomous paths using planes detected by vision sensors. In addition, studies on an optimization-based path planner approach are being conducted [6,7].

Path planning is largely divided into global path planning and local path planning. Global path planning aims to find the optimal path rather than the time to compute the path. On the other hand, local path planning aims to quickly recognize nearby obstacles and find a path to move safely using sensors. The important thing in the global path planning algorithm is to search an optimal path to satisfy various conditions. For example, like the Dijkstra algorithm [8], it may be to find a path that minimizes the distance between the starting point and the destination, and if the cost is selected as the difference in the angle or height of the foot between the previous and the current step, the result of the path may change accordingly. Some teams participating in the DARPA Robotics Challenge (DRC) Finals used planners to search for a path from the robot’s current location to the mission area related to disaster response [9]. For example, Team VIGIR used Anytime Repair A* (ARA*) planning method to find footsteps that considered shortest paths [10]. In addition to ARA* planning, there were A*-based Footstep planners that created the optimal footpath to move the robot to the desired goal. Joel Chestnutt et al. have proposed an A* search two-dimensional footstep planner that moves to the goal while avoiding obstacles [11]. Philip Michel et al. have installed a camera 3.5 m above the ground to show an environment map and presented an approach to autonomous humanoid walking via the A* planner [12]. Philip Karkowski et al. have presented a planner combining Adaptive 3D Action Set and A* to efficiently find valid footstep paths in real-time [13]. Dimitrios Kanoulas et al. have proposed a new footstep planner that incorporates curved patch contact analysis to handle even non-flat curved terrain [14]. IHMC has proposed a new A* footstep planner that allows partial footholds to increase the number of footholds available after decomposing the environment into a flat area [15]. These planners convert the obstacle information on the map into a graph composed of nodes and edges. In addition, it uses a cost function combined with a heuristic, and when the optimal path is determined, it is expressed as a series of footsteps afterward.

The global path planning algorithm searches for the shortest path to the goal but requires pre-calculation, so it takes a very long time to calculate the path if the distance to the goal is long or there are many obstacles. In addition, it can be used only when information on the global map is secured through a distance measuring sensor such as LIDAR. In other words, these planners are useless unless mapping is performed when a robot is introduced to an unfamiliar environment. Studies on local planners that generate footprints within a limited area acquired via a camera without the global map have been performed. Kei Okada et al. have segmented planes using the three-dimensional (3D) Hough transform and created 3D footprints along a straight line [16]. Their planner places the footsteps as far as possible while the robot is in a planar area. When the robot encounters an obstacle, it creates footprints to place it close to the front of the obstacle and then steps over it. Cupec et al. have updated a local environment map using information about obstacles detected through a camera [17]. This map classifies obstacles by contours obtained via connecting the boundaries of the walking area and edges of the obstacles. In the walking area, the robot’s footsteps walk straight and cross obstacles, each of which has a fixed length of steps. M. Yagi and V. Lumelsky have set normal steps of a fixed length and set each step of a predefined length to walk when the distance between the robot and the obstacle is whole, half, and a quarter of a normal step [18]. These local planners tend to cause robots to fall into the local minima on the way to the goal because they walk in a fixed direction or according to walking patterns of a fixed length. If the robot falls into the local minima, it may be slow to search for a path to reach the goal or find an undesired path.

There is an algorithm named a rapidly exploring random tree (RRT) that quickly generates a feasible path as a way to avoid local minima [19]. This algorithm randomly generates points in the search space and grows a tree from the starting point to find a path that reaches the destination. However, RRT is a method of a global path planning algorithm, which has the disadvantage that it can be applied only by knowing the information of obstacles and the destination in the global map. Therefore, there is a need for a path planning algorithm that finds a path that a humanoid robot can stably walk even in an environment without a global map.

This paper presents a strategy for placing footsteps in a narrow field of view through a vision sensor. Through this strategy, the legged robot calculates the position of the footsteps to stably cross objects. While the robot is walking, objects may not be detected due to the narrow camera field of view. In addition, even if the landing area is found, a collision between the legs of the robot may occur. A robot-mounted vision PC automatically calculates the foot position and direction for the robot’s walk as well as the swing foot selection based on the detected plane within a limited area, and stores a history of both feet while the robot is walking. Existing A*-based footstep planners do not generate a path if there is no solution to reach the goal due to the completeness of A* and are vulnerable to change in the object position if the map is not updated during path movement. Unlike A*-based footstep planner, our strategy computes footsteps that the robot can walk on without obtaining a global map. We find a path that allows us to quickly recognize and move around objects by updating the map every walking step. The robot may not be able to detect objects due to its limited field of view. In addition, even if an area to land can be found, collision between the legs of the robot can occur. Our strategy used the previous landing point data to solve this problem. The contribution of this paper is to create a footstep through vision sensor information in a narrow field of view without global map and to find a path to move stably by continuously updating information on cinder blocks. In addition, even if an area to land can be found, there may be situations in which the robot’s legs collide. Our proposed strategy can solve this problem by using previous landing point data. This study is organized as follows: Section 2 describes the process of detecting the plane of an object, and Section 3 presents a strategy for moving while looking at a limited area. Section 4 describes the hardware configuration and system architecture of the RoK-3 robot used in the experiment. Section 5 describes the experimental results for our proposed strategy.

## 2. Plane Detection

**Figure 1:**
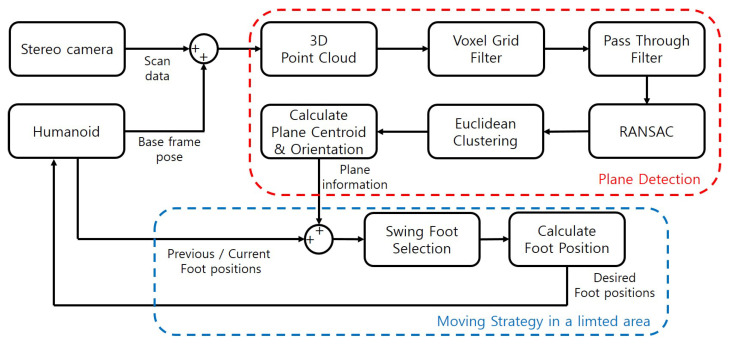
Pipeline of footstep planner.

Figure 1 is the pipeline of the footstep planner that shows the strategy of moving while looking at a limited area. The robot must detect the plane of an object to cross it; thus, we obtain point cloud data of a limited area via a stereo camera attached to the robot.

A point cloud represents a set of a series of points, each containing the position of the 3D coordinate system. In plane detection in Figure 1, Voxel Grid Filter, Pass Through Filter, and Euclidean clustering techniques are commonly used techniques for point cloud processing [20]. We reduced computational time for plane detection by filtering through these techniques. We then detect the planes for the objects from the filtered point cloud and calculate the center point position and orientation of each plane. Using the calculated plane information, the robot could determine the foot position and walking direction.

### 2.1. Point Cloud Filtering

**Figure 2:**
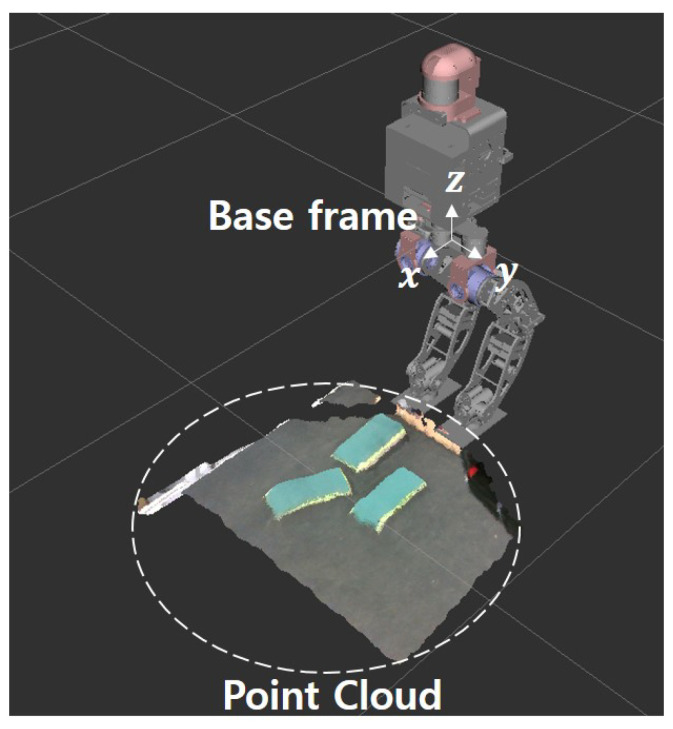
Point cloud with coordinates transformed based on the base frame.

A point cloud requires filtering to reduce the computational burden of its large amount of data. To reduce the computational load, we use the following filters:

- Voxel grid filter using an octree: The purpose of this filter is to reduce the computational load by reducing the number of points. After placing the same length of cubes in the point cloud at regular intervals, the centroid for all points existing inside each cube is calculated. After the calculation, the number of point clouds is reduced by removing the remaining points, except for the calculated centroid. It is important to quickly find the points that are configured to process the point cloud, so we use an the octree structure to quickly search for the points. An octree is a hierarchical data structure for 3D spatial division that divides into eight volumes until it reaches a cube with a specific resolution length [21].
- Transformation: The point cloud acquired from the camera is measured on the basis of the camera’s coordinate system and needed to be checked intuitively. Therefore, we recalculate the position value of the point cloud by transforming the coordinates based on the base frame representing the robot (Figure 2).
- Pass through filter: This is a filter that passes only the points that are in the area of interest and removes those that are not in the area. We reduce the computational load by specifying a range for each axis on a 3D space and using only the points within the specified coordinate range.

### 2.2. Random Sample Consensus (Ransac)

We use RANSAC to detect planes for the robot to walk on. This is a method of iterating to estimate the parameters of the desired mathematical model from a data set containing an outlier that prevents the estimation of model parameters [22].

- Parameter: Sampling iterations and distance thresholds are required when using RANSAC. The distance threshold is the value of how to set the boundary between the inlier and outlier.
- Plane normal: We restrict the robot from walking on a plane with a large slope such as a roll or pitch. The normal vector of the plane is obtained through plane coefficients a, b, c, and d calculated using RANSAC. Then, the angle between the normal vector of the plane and the z-axis unit vector (0, 0, 1) is calculated based on the reference frame shown in Figure 2. If the angle between the two vectors is more than 15°, the robot considers it difficult to walk and excludes the plane.

### 2.3. Euclidean Clustering

**Figure 3:**
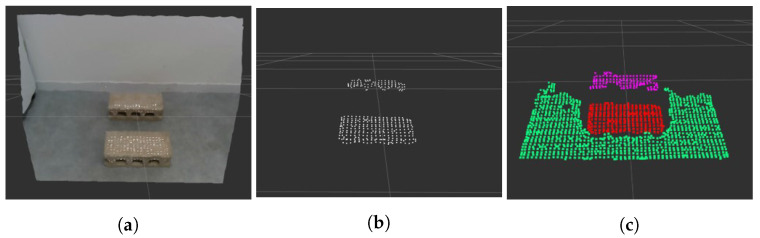
Point cloud results for each method. (**a**) Original; (**b**) RANSAC; (**c**) Euclidean clustering.

Once a plane is detected through RANSAC, we have to divide it because it is considered to be the same plane shown in Figure 3b. Using the Euclidean clustering technique, if the distance between two points in the point cloud is below a certain threshold, the points are included in the same group to split the planes.

### 2.4. Calculating the Centroid and Orientation

**Figure 4:**
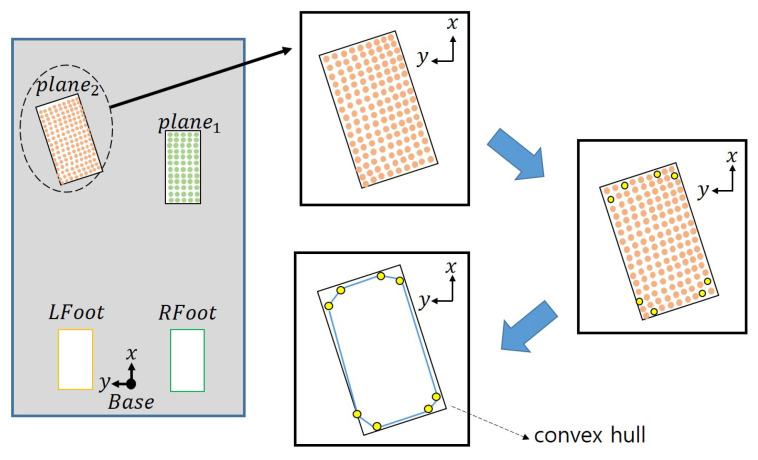
Points that make up a convex hull based on a given point cloud.

**Figure 5:**
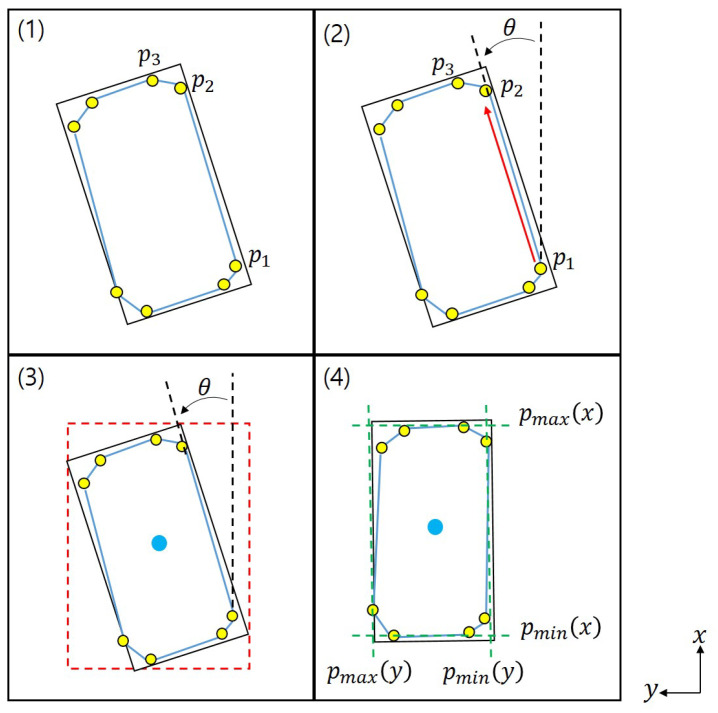
The method for determining the orientation of a plane through a rectangle with minimum area. (**1**) Points constituting the convex hull; (**2**) Angle calculated from points; (**3**) Axis-aligned bounding box; (**4**) Bounding box with minimum area.

The center position and orientation for each plane are required to apply our proposed footstep planning. The center position is calculated from the average value of the points corresponding to each plane. Next, we calculate the distance between the robot’s base frame and the centroid and arrange the planes in the order of small distances. We exploit vertices for the convex hull to compute each plane’s orientation. As shown in Figure 4, we construct the convex hull in the point cloud and obtain the positions of the points that constitute it. In the Figure 4, Base means the base frame of the robot, and LFoot and RFoot mean the left and right feet of the robot. The obtained convex hull construction points are indicated by yellow dots in Figure 4 and Figure 5. After that, create an Axis-Aligned Bounding Box (AABB) surrounding these points as shown in Figure 5(3), and calculate how much rotation must be made based on the center point to become an Oriented Bounding Box (OBB) using the formula (1) below.

(1) $$\begin{array}{c} \theta =tan^{-1}\left(\frac{p_{i+1}\left(y\right)-p_{i}\left(y\right)}{p_{i+1}\left(x\right)-p_{i}\left(x\right)}\right) \end{array}$$

In Figure 5, *p* denotes the points constituting the convex hull. Figure 5(4) shows the bounding box when the calculated angle is rotated in the opposite direction. In this case, the bounding box is the area of the rectangle using the minimum and maximum values of each axis among the convex hull construction points. This area is the smallest area and this angle is determined in the direction of the plane.

## 3. Moving Strategy in a Limited Area

**Figure 6:**
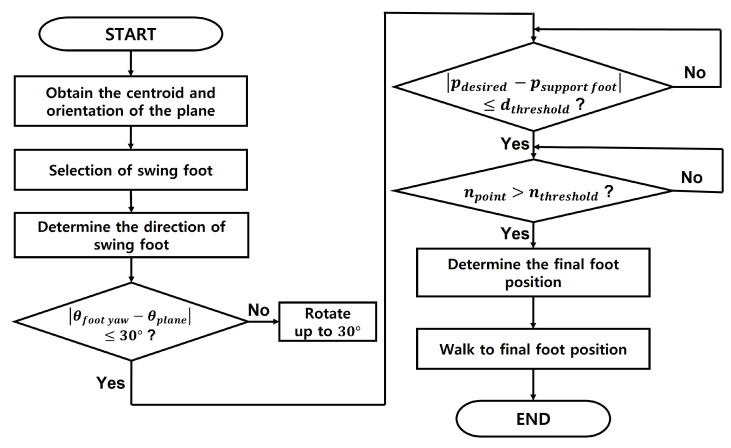
The flow chart of the moving strategy.

Here, we present a strategy to move from a limited area using a sensor even if we do not know the surrounding map. This strategy uses the plane information to calculate where the robot should go. Figure 6 shows the flow chart of the moving strategy, and the steps for selecting the position of the foot are as follows.

1. Calculate the center point and orientation of each plane by the method suggested in Section 2.4.
2. Check the y-axis coordinate value of the center point of the first plane among the planes determined in the order closest to the base frame. Depending on which side this value is based on the base frame, the swing foot is determined as the left foot or the right foot.
3. The yaw direction of the swinging foot is defined as the yaw value of the first plane. If the difference between the direction of the swing foot and the direction of the current foot exceeds a specific threshold value (e.g., 30 degrees), the value is changed to maintain as much as a specific threshold value.
4. To determine the maximum distance the foot can travel, check that the difference between the center point of the support foot and the swing foot exceeds a certain threshold.
5. Draw a rectangle with the size of the foot centered on the points obtained in step 4. A point in which the number of overlapping points between the drawn rectangle and the detected plane area satisfies a value greater than a specific threshold value is found.
6. Among the points satisfying the above conditions, the point with the largest number of overlapping points is determined as the final point to which the swing foot should go. The robot walks to this point. If there are two or more points with the same maximum number, the point with the greatest maximum distance is selected.

**Figure 7:**
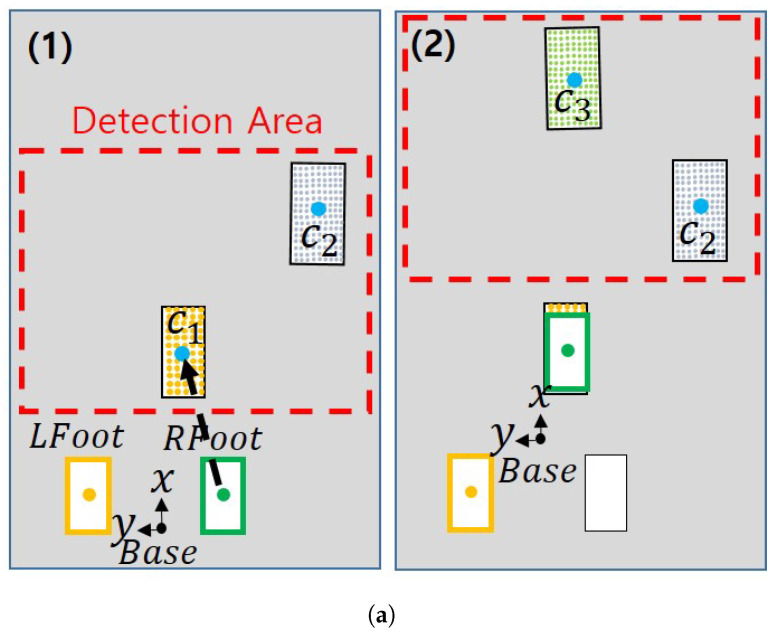

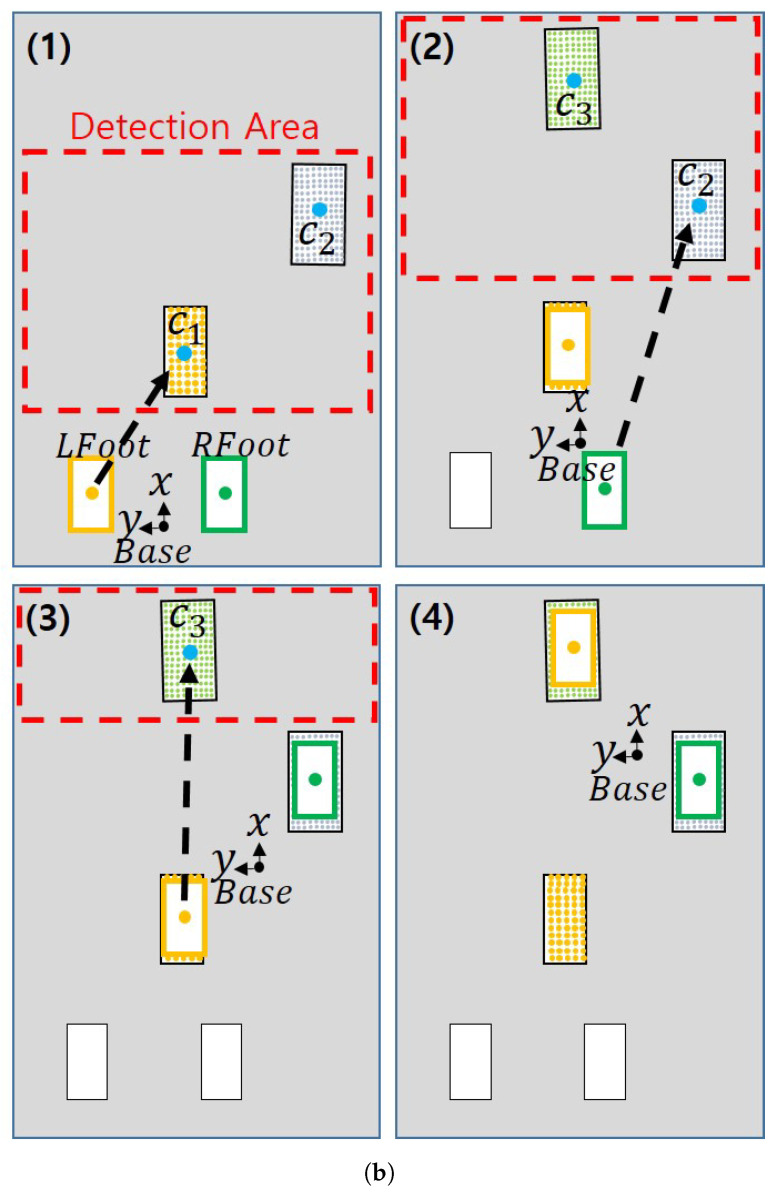
When the first step swing foot is the left foot, the result of reaching the goal. (**a**) (**1**,**2**) mean the situation in which each footstep moves. (**b**) (**1**–**4**) mean the situation in which each footstep moves.

Figure 7 shows that the goal that can be reached varies depending on which swing foot the robot uses. This is because a path to reach the goal cannot be found due to a collision between the legs. In Figure 7, the blue dot and *c* indicate the center point of the plane. If the right foot is the swing foot, such as in Figure 7a(2), there is an occurrence of singularity due to the distance between two feets, and if the left foot is the swing foot, there may be a collision between the legs. Therefore, we describe a method to escape from these situations.

**Figure 8:**
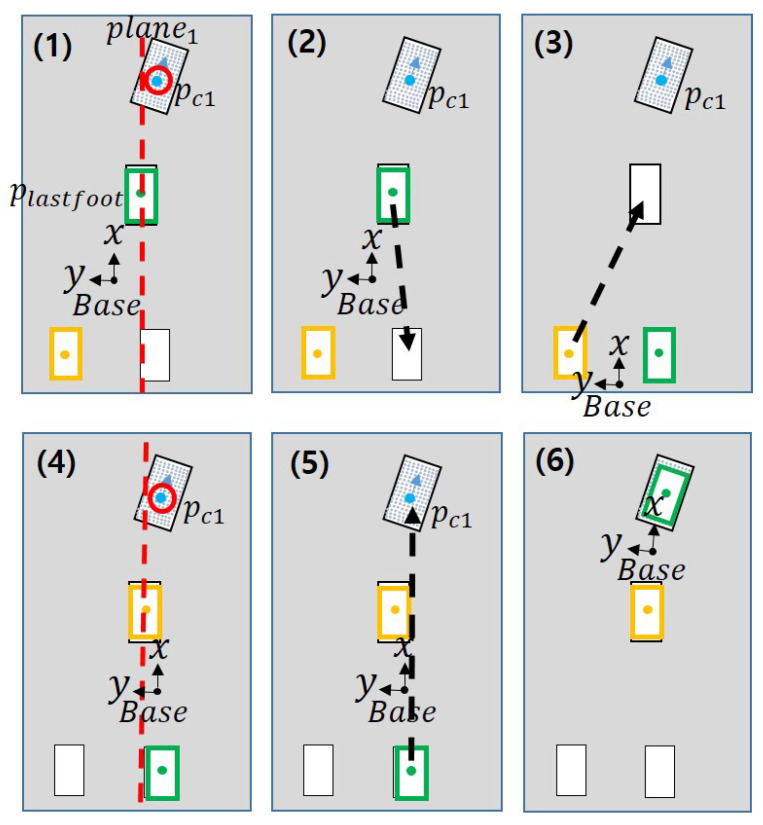
Results when the last swing foot and the swing foot calculated in (1) are the same. (**1**–**6**) are the process of selecting footstep and moving.

**Figure 9:**
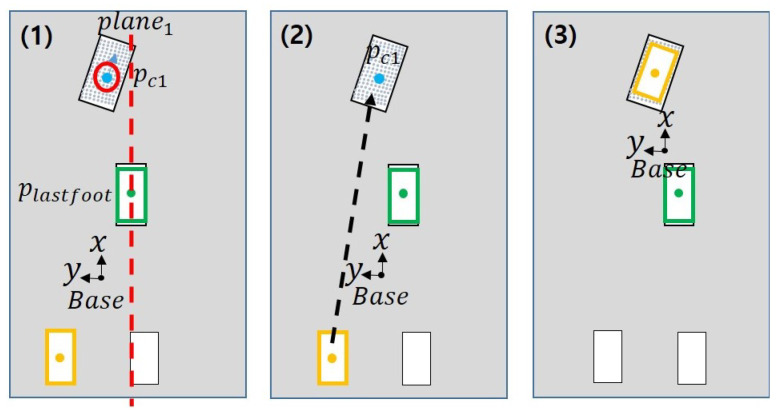
Results when the last swing foot and the swing foot calculated in (1) are different. (**1**–**3**) are the process of selecting footstep and moving.

To start, the robot uses the plane centroid to determine the swing foot to select. Even if a plane to walk on is found, when the robot cannot move, move the last swing foot to the previous position for that foot. Subsequently, this problem is solved by placing the other foot other than the swing foot in the position of the last swing foot that was before it moved to the previous position. The steps to select the swing foot are as follows:

1. Compare the position of the last swing foot with the position of the first plane centroid, as shown in Figure 8(1). If the center point of the plane based on the y-axis is to the left of the position of the last swing foot, set the next swing foot as the left foot.
2. Make sure that the last swing foot and the swing foot calculated in Step 1 are the same foot.
3. If the last swing foot and the calculated swing foot are the right foot (Figure 8), the last swing foot returns to the previous position while the next swing foot is replaced by the other foot and moves to the same position.
4. If not the same (Figure 9), the next swing foot is determined by the calculated swing foot.

### Selecting the Proper Stepping Foot Position on a Plane

The yaw angle of the stepping foot is determined by the plane angle obtained by the plane detection. At this time, if the difference between the current yaw angle of the robot’s foot and the angle value of the plane is over a specific threshold value, it is rotated up to a threshold to prevent excessive rotation. After determining the direction of the swing foot, it calculates an appropriate position to move through the detected plane. If the robot’s feet are too far apart, singularity can occur, and if the plane area to be stepped on by the robot is small, the robot is likely to fall. To address this, we determine the distance threshold between the robot’s support foot and swing foot to find points only within the threshold. Sylvain Bertrand et al. allow their robot to walk on partial footholds to increase the number of footholds available to it [23]. Inspired by this algorithm [23], we let our robot walk on partial footholds by searching for available footholds according to the number of point clouds.

**Figure 10:**
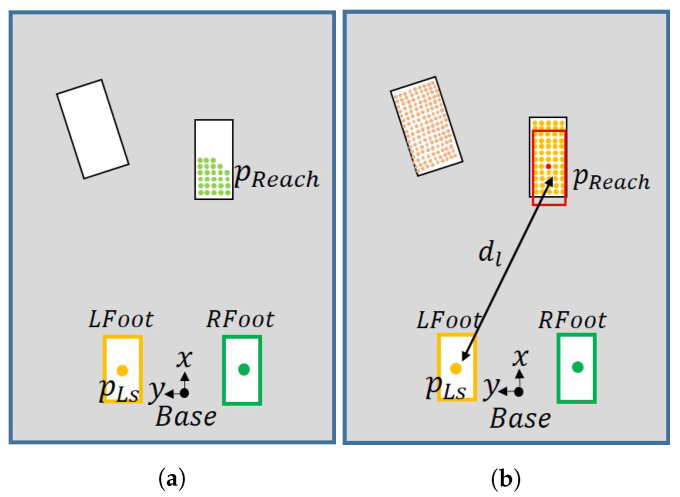
Algorithm for determining foot position using distance and overlapping area. (**a**) Points within the maximum distance; (**b**) Rectangle the size of foot around the point.

As shown in Figure 10a, we determine the maximum distance the foot can move and find points within the maximum distance. The light green dots in the Figure 10a indicate points within the maximum distance. The location marked LS is the center point of the left foot, that is, the supporting foot. Then, we draw a rectangle the size of the robot’s foot around the point, as shown in Figure 10b. If the number of points in the area is less than a certain threshold, the point is excluded. Among the remaining points, we find one point with the largest number of points in the area and set it as the final foot position of the robot. If there are more than two points with the largest number of points, we select that with the largest distance between the swing foot and support foot. If no planes are found or there is no suitable stepping foot position, the search is terminated.

## 4. An Overview of Rok-3

### 4.1. Hardware of Rok-3

**Figure 11:**
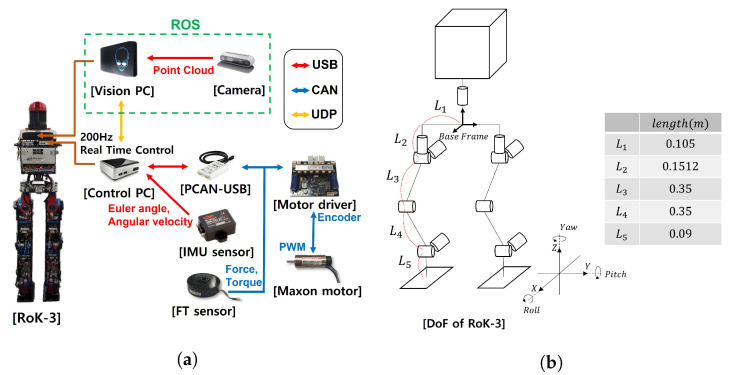
(**a**) The communication system about RoK-3; (**b**) Degree of Freedom about RoK-3.

As shown in Figure 11, we used biped robot RoK-3 to verify the strategy for moving while looking at a limited area in a real environment. The robot’s PC comprised a vision PC for image processing and a control PC for walking. Maxon BLDC and Faulhaber motors and Harmonic Drive gears were used. Several sensors were installed to recognize the robot’s status. An FT sensor was located at the end of each ankle, and an IMU sensor and stereo camera were attached to the pelvis. The stereo camera used Intel’s Realsense d435i camera.

The kinematic information of RoK-3 is explained in Figure 11b. In general, humanoid robot walks with their knees bent to avoid the singular problem when walking. RoK-3 walks with the height of the Center of Mass (CoM) lowered by 52.5 cm. To prevent the occurrence of singular kinematically, the distance in the x-y plane of the sole for the hip joint of each leg was limited to 30 cm. Therefore, in this study, the distance between the two feet did not exceed 60 cm.

### 4.2. System Architecture

The robot’s vision PC used Ubuntu OS and the Robot Operating System. The control PC used Ubuntu OS and the Xenomai Real-Time Operating System for real-time control at 200 Hz. Xenomai is a development framework used to create real-time threads in a Linux environment [24]. The motor controller received a value for the target angle from the control PC at the same cycle via controller area network (CAN) communication. The control PC obtained the motor’s encoder information from the motor controller and the FT sensor data. The IMU sensor used USB communication and received data at 200 Hz.

## 5. Experiments

For stabilizing the robot’s walking, our control algorithm used the controller developed in the previous study [25,26]. To prove the proposed algorithm, we describe three experiments consisting of cinder blocks. The width, length and height of the cinder block were 0.23 m, 0.115 m, and 0.06 m, respectively, and the cinder blocks were placed at the same height in the three experiments. For the robot to move to the desired destination, a combination of walking straight and turning is required. In addition, even if an area for the robot to land is found, a situation in which the robot’s legs collide may occur. Considering these situations, we placed the cinder blocks differently in each experiment. The robot autonomously calculates where to step on the cinder block and moves according to each situation. Since some time is needed to calculate the position of the footstep placement, our robot walked statically at a slow speed. Through the experiment, it was confirmed that the robot walks stably in three environments. The robot can walk most paths by combining the configurations of these environments. Experimental video can be found at Supplementary Materials.

### 5.1. Scene 1: Walking Straight

**Figure 12:**
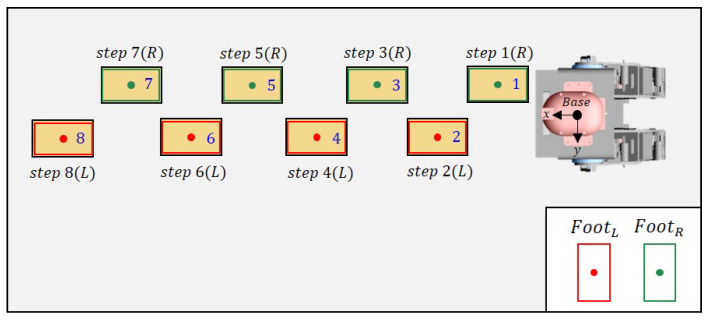
Top view of Scene 1.

In this experiment, the cinder blocks are laid out in a straight line. Each cinder block is placed at approximately 0.25 m intervals. Figure 12 shows the robot’s footsteps (red, green) and cinder blocks (yellow) when looking at the robot from above. The step indicates the footstep order in which the robot walked and the swing foot at this time. The robot calculates the center point and direction of the plane based on the detected plane. After that, the detected planes are sorted in ascending by calculating the distance between the robot’s base frame and the center point of the plane.

**Figure 13:**
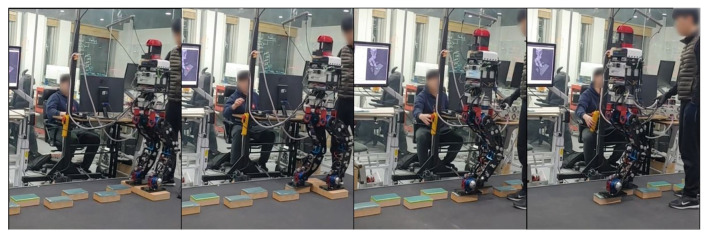
The experiment of Scene 1.

Figure 13 shows the actual robot walking in Scene 1. First of all, the closest plane to the base frame was on the right side of the base frame, so the right foot was set as the swing foot. Then, the robot walked by calculating the appropriate position through the proposed strategy, as shown in Figure 1.

**Figure 14:**
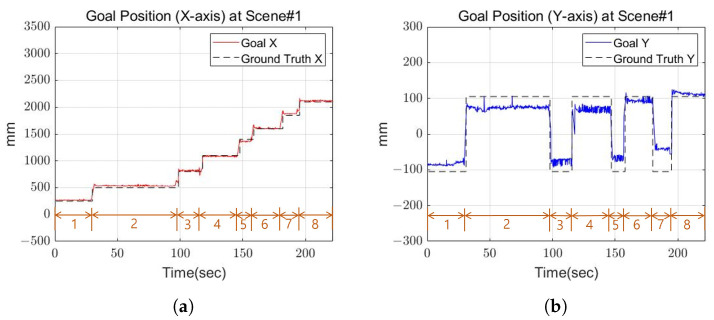
The goal position of Scene 1. (**a**) X-axis goal position at Scene 1; (**b**) Y-axis goal position at Scene 1.

**Figure 15:**
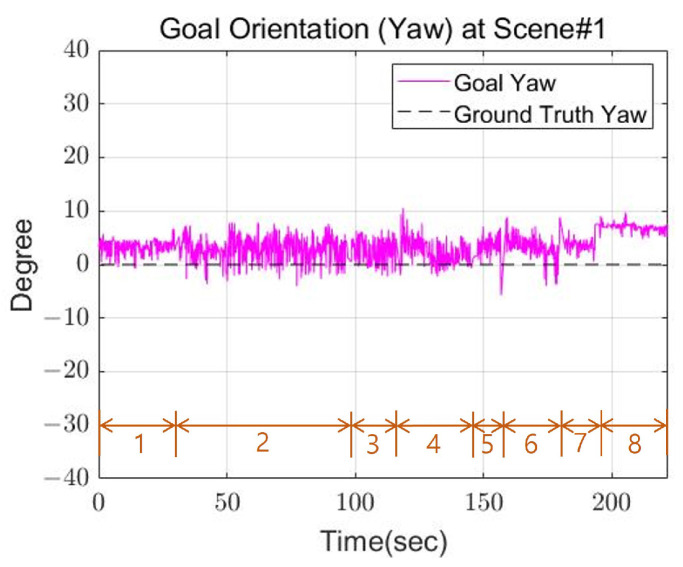
The goal orientation of Scene 1.

Figure 14 shows the goal position calculated by the robot and the actual position of the cinder block. In the graph, the orange number indicates the step for the robot’s next footstep. Figure 14 and Figure 15 show the calculated position and the actual position of the cinder block in the actual experiment. Figure 15 shows the yaw calculated by the robot, and the position and orientation recalculate the goal position and orientation for the next step after walking one step. From the results, the x-axis and y-axis average errors for the two values occurred within 4 cm, and the angular average error came out to within 5 degrees. Through this, we confirmed that the calculated location is similar to the actual position of the cinder block.

### 5.2. Scene 2: Replanning

**Figure 16:**
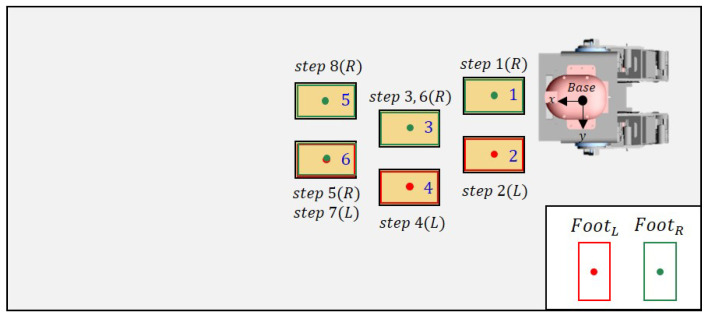
Top view of Scene 2.

This experiment is an experiment in which the robot walks by replanning its path when it is unable to walk in the face of a specific situation. In this experiment, even if an area to land is found, a situation may occur where the robot’s legs collide. Figure 16 shows the robot’s footsteps (red, green) and cinder blocks (yellow) when looking at the robot from above. The closest plane to the base frame was on the right side of the base frame, so the right foot was set as the swing foot.

**Figure 17:**
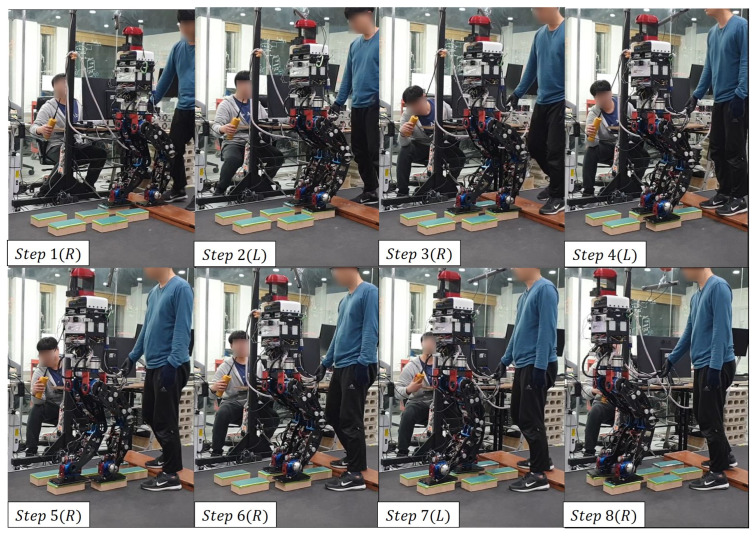
The experiment of Scene 2.

When the robot’s right foot arrives at the position of the sixth cinder block in step 5 of Figure 17, the robot’s left foot cannot be placed in the fifth cinder block due to a robot’s leg collision. To solve this problem, the robot uses the previous footstep position to move backward and then replan the path. Steps 7 and 8 shown in Figure 17 show the replanned path movement.

**Figure 18:**
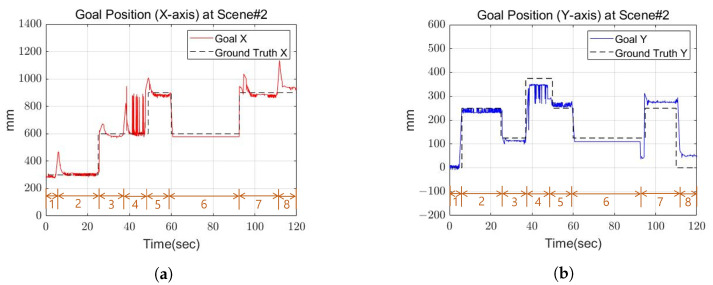
The goal position of Scene 2. (**a**) X-axis goal position at Scene 2; (**b**) Y-axis goal position at Scene 2.

**Figure 19:**
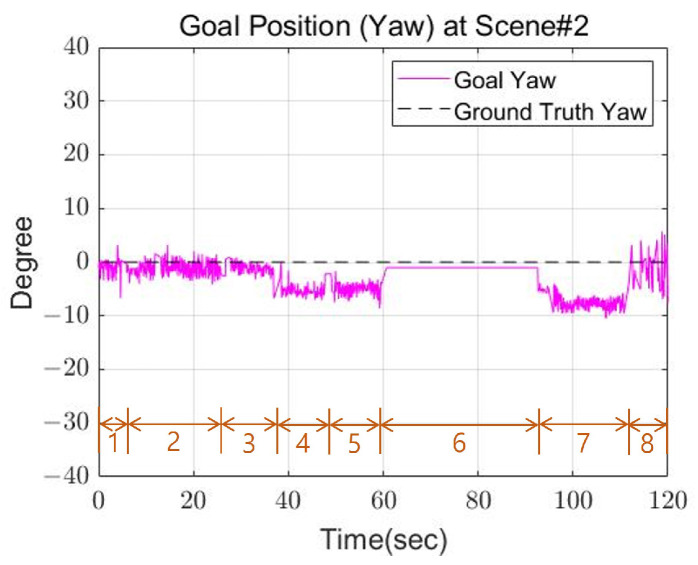
The goal orientation of Scene 2.

Figure 18 and Figure 19 shows the calculated position and the actual position of the cinder block. In the step 6 section of the Figure 18a, the x-axis position decreased, which means that the last swing foot was calculated to return to its previous position to solve the robot’s leg collision. From the experimental results, mean position error between actual and calculated positions occurred within 3 cm, and the average angle error occurred within 4 degrees.

### 5.3. Scene 3: Turning

**Figure 20:**
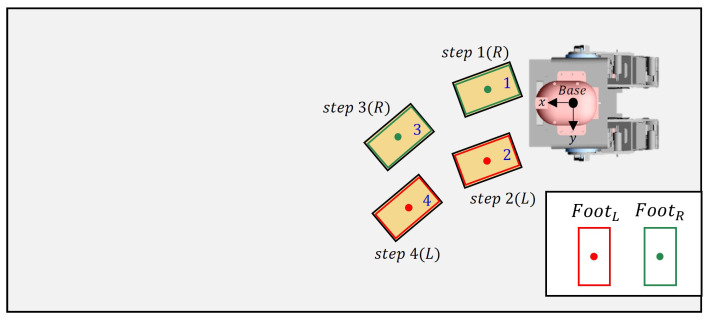
Top view of Scene 3.

**Figure 21:**
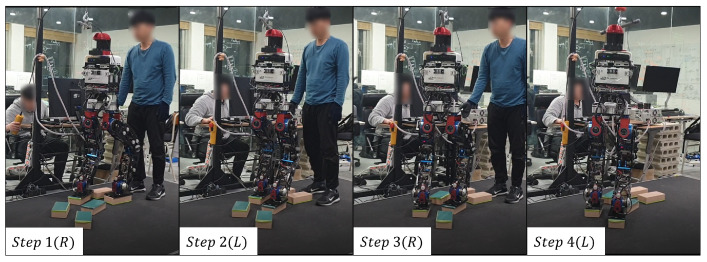
The experiment of Scene 3.

**Figure 22:**
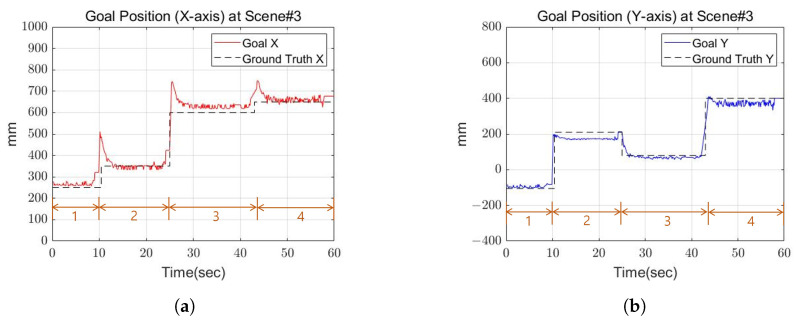
The goal position of Scene 3. (**a**) X-axis goal position at Scene 3; (**b**) Y-axis goal position at Scene 3.

**Figure 23:**
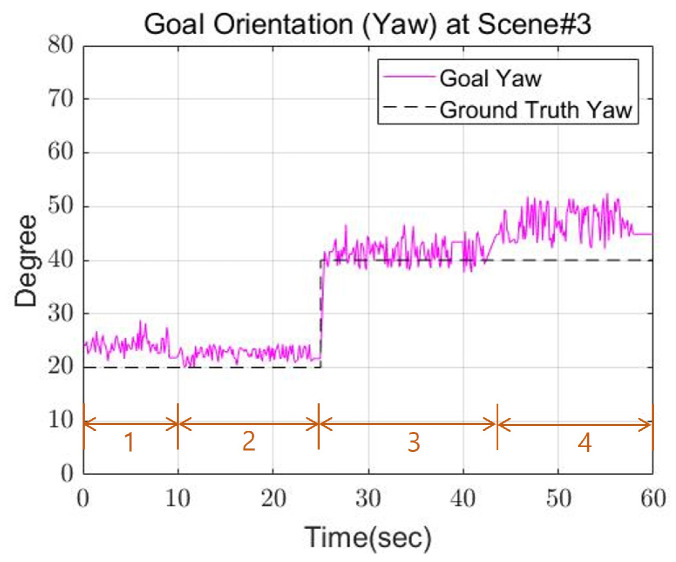
The goal orientation of Scene 3.

Figure 20 shows the footsteps and cinder blocks the robot walked when looking at the robot from above. In this experiment, the cinder blocks are laid out in a curve shape. The rotation angle interval of the cinder block was set based on 20 degrees. Figure 21 shows the actual robot walking in Scene 3. First of all, the closest plane to the base frame was on the right side of the base frame, so the right foot was set as the swing foot. In this case, the angle of the plane represents an angle required to become a rectangle having a minimum area. In the experiment, if the difference between the angle of the foot to swing and the angle of the plane was greater than a certain threshold, restrictions were applied. Then, calculate the distance between the supporting foot and the foot to swing and check whether it exceeds a certain threshold. Finally, an area equal to the size of the robot’s foot is created centered on one point, and the point with the most overlapping points with the plane is set as the final foot position of the robot. Figure 22 and Figure 23 show the calculated position and the actual position of the cinder block. From the experimental results, mean position error between actual and calculated positions occurred within 4 cm, and the average angle error occurred within 5 degrees.

## 6. Conclusions

A recent study clusters point cloud data obtained from sensors into planar regions to detect areas that a legged robot can walk in in a complex environment [23]. With the planar area obtained through these algorithms, the robot automatically calculates a footstep planning solution. This planner defines an A*-based footstep node containing foot position and orientation information and finds a solution through a series of steps (e.g., node expansion, node snapping, edge inspection, and edge scoring) [15]. The more considerations and planning scope, the more time these plans will take to find a solution. we propose a strategy for the legged robot to stably walk with limited camera information in an environment where information about the surrounding environment is unknown. In the local planning method, the robot tends to fall into the local minimum value, so we proposed a method to solve this by using the position of the robot’s previous footsteps. To calculate the position and direction of the robot’s footsteps, the maximum travel distance from the supporting foot was considered. Moreover, to increase the number of available footholds, partial footholds were allowed, and the angle of the foot that the robot could rotate was limited to prevent excessive rotation. Finally, it was confirmed that the robot automatically calculated the proper foot position and walked in situations comprising various patterns.

Our future work will involve determining the appropriate position and orientation for objects of different heights, and based on this work, we will conduct a study in which the biped robot recognizes planes and generates footsteps on stairs.

## Data Availability

Not applicable.

## Supplementary Materials

The following supporting information can be downloaded at: https://www.mdpi.com/article/10.3390/s22103817/s1, Video S1: Experiment Video.
